# Prospective Evaluation of a Modified Apnea Test in Brain Death Candidates that Does Not Require Disconnection from the Ventilator

**DOI:** 10.1007/s12028-024-02035-w

**Published:** 2024-07-01

**Authors:** Johann Lambeck, Jürgen Bardutzky, Christoph Strecker, Wolf-Dirk Niesen

**Affiliations:** Department of Neurology and Clinical Neurophysiology (Klinik für Neurologie und Neurophysiologie), Freiburg University Medical Center (Universitätsklinikum Freiburg), Breisacherstr. 64, 79106 Freiburg, Germany

**Keywords:** Irreversible loss of brain function, Apnea test, Brainstem areflexia, Ventilator-based, Apneic oxygenation

## Abstract

**Background:**

The apnea test (AT) is an important component in the determination of brain death/death by neurologic criteria (BD/DNC) and often entails disconnecting the patient from the ventilator followed by tracheal oxygen insufflation to ensure adequate oxygenation. To rate the test as positive, most international guidelines state that a lack of spontaneous breathing must be demonstrated when the arterial partial pressure of carbon dioxide (PaCO_2_) ≥ 60 mm Hg. However, the loss of positive end-expiratory pressure that is associated with disconnection from the ventilator may cause rapid desaturation. This, in turn, can lead to cardiopulmonary instability (especially in patients with pulmonary impairment and diseases such as acute respiratory distress syndrome), putting patients at increased risk. Therefore, this prospective study aimed to investigate whether a modified version of the AT (mAT), in which the patient remains connected to the ventilator, is a safer yet still valid alternative.

**Methods:**

The mAT was performed in all 140 BD/DNC candidates registered between January 2019 and December 2022: after 10 min of preoxygenation, (1) positive end-expiratory pressure was increased by 2 mbar (1.5 mm Hg), (2) ventilation mode was switched to continuous positive airway pressure, and (3) apnea back-up mode was turned off (flow trigger 10 L/min). The mAT was considered positive when spontaneous breathing did not occur upon PaCO_2_ increase to ≥ 60 mm Hg (baseline 35–45 mm Hg). Clinical complications during/after mAT were documented.

**Results:**

The mAT was possible in 139/140 patients and had a median duration of 15 min (interquartile range 13–19 min). Severe complications were not evident. In 51 patients, the post-mAT arterial partial pressure of oxygen (PaO_2_) was lower than the pre-mAT PaO_2_, whereas it was the same or higher in 88 cases. In patients with pulmonary impairment, apneic oxygenation during the mAT improved PaO_2_. In 123 cases, there was a transient drop in blood pressure at the end of or after the mAT, whereas in 12 cases, the mean arterial pressure dropped below 60 mm Hg.

**Conclusions:**

The mAT is a safe and protective means of identifying patients who no longer have an intact central respiratory drive, which is a critical factor in the diagnosis of BD/DNC.

*Clinical trial registration* DRKS, DRKS00017803, retrospectively registered 23.11.2020, https://drks.de/search/de/trial/DRKS00017803

## Introduction

The apnea test (AT) determines whether a loss of spontaneous respiration has occurred and serves as an important clinical component of all relevant international guidelines for the determination of brain death/death by neurologic criteria (BD/DNC) [1–5]. The consensus of many of these guidelines is that to show central respiratory failure, there must be evidence of spontaneous breathing failure when arterial partial pressure of carbon dioxide (PaCO_2_) is at a level of at least 60 mm Hg. Some guidelines also require an increase in PaCO_2_ of at least 20 mm Hg above a known elevated baseline (and parallel pH drop to values < 7.30) and deem this to be consistent with spontaneous breathing failure [1, 3, 5, 6].

The corresponding German guideline recommends that after excluding a relevant adaptation to an increase in PaCO_2_ (e.g., chronic in the context of chronic obstructive pulmonary disease or acute in patients with pulmonary contusion) and by starting at a baseline PaCO_2_ of 35–45 mm Hg, hypercapnia should be induced by acute hypoventilation until the aforementioned PaCO_2_ value of 60 mm Hg is reached. Constant clinical monitoring should be performed in parallel to detect respiratory excursions or spontaneous respiratory efforts by the patient. Hypoxia must be avoided, and sufficient oxygenation of the patient, such as by intratracheal O_2_ insufflation, is mandatory. Disconnection of the patient from the ventilator is often performed to assess the failure of spontaneous breathing [1, 2, 6]. It is mandatory that preserved patient-generated breaths can be unambiguously distinguished from ventilator/cardiac oscillations, the latter of which can generate an apparent breath (“auto-triggering”) and hence lead to false labeling of an otherwise preserved ventilatory drive [7].

Because many of the candidates for BD/DNC have pulmonary impairment (PI) due to conditions such as aspiration pneumonia, neurogenic pulmonary edema, and/or acute respiratory distress syndrome (ARDS) [8–10], a relevant proportion of these patients is dependent on both high positive end-expiratory pressure (PEEP) values and an increased fraction of inspired oxygen (FiO_2_) for pulmonary stabilization. The loss of PEEP upon disconnection from the ventilator can render the AT very difficult or even impossible. The risk of hypoxia and associated cardiopulmonary instability, including the need for resuscitation, has been previously described [11]. De-recruitment and hypoxemia have been reported with variable frequency, including in patients on extracorporeal membrane oxygenation (ECMO) [12, 13].

Therefore, various methods have been described for performing the AT in a more protective manner in this patient group [9, 14–16]. One of the methods described in retrospective studies and case series/reports is an AT that is conducted under continuous positive airway pressure (CPAP) and sometimes ensues without disconnection from the ventilator [17–20]. The aim of this study was to prospectively investigate whether a modified version of such an AT that is performed while the patient is connected to the ventilator (i.e., without disconnection) is safe, feasible, and applicable to a larger number of patients, especially those with impaired pulmonary function. Furthermore, this study aimed to identify any potential difficulties arising from the application of this test.

## Methods

### Ethics Check

This study was approved by our local ethics committee (Nr. 368/19) and registered at the German Clinical Trials Register (DRKS00017803). In all cases, informed consent was obtained from the patients’ relatives. No external funding was received.

### Study Population

A group of 140 patients with severe brain damage of various etiologies (Table 1) were consecutively enrolled between January 2019 and December 2022 and examined for the presence of BD/DNC. Patients were examined for the presence of BD/DNC at various intensive care units within the University Medical Center Freiburg.

**Table 1:** Depiction of patient characteristics of all 140 included patients and further details of the BD examinations

	Value
Age	Median 56 years (IQR 44–67.5)
Sex [female/male]	58/82
Included patients	140
mAT initiated	139^a^
mAT aborted	0
mAT result	
No spontaneous respiration (mAT positive)	127
Spontaneous respiration preserved (mAT negative)	12
Duration of mAT	Median 15 min (IQR 13–19)
Etiology of brain lesion	
ICH	23
SAH	12
Ischemic Stroke	16
TBI	4
Hypoxia	40
Combined^b^	37
Other	8
Patient on vaECMO/ECLS	16
Relevant levels of analgesics/sedatives	9^c^
Relevant COPD^c^	5^c^
No PI	45 (32%)
PI grade 1	17 (12%)
PI grade 2	46 (33%)
PI grade 3	31 (22%)
BD/DNC confirmed	114
BD/DNC not confirmed	26

^a^One patient was deemed to be too unstable to initiate the AT (fraction of inspired oxygen FIO_2_ 100%, positive end-expiratory pressure PEEP 10 mbar [7.5 mm Hg], oxygen saturation (SpO_2_) 90%, arterial partial pressure of oxygen (PaO_2_) 75 mmHg)

^b^Combined = A combination of two or more different etiologies, e.g., SAH and cardiopulmonary resuscitation (CPR)-related hypoxia, TBI and ICH, or SAH, etc.

*AT* apnea test, *BD/DNC* “brain death/death by neurologic criteria” (irreversible loss of whole brain function), *COPD* chronic obstructive pulmonary disease, *ECLS* extracorporeal life support, *ICH* intracerebral hemorrhage, *IQR* interquartile range, *mAT* modified apnea test, *PI* pulmonary impairment, *SAH* subarachnoid hemorrhage, *TBI* traumatic brain injury, *vaECMO* veno-arterial extracorporeal membrane oxygenation

^c^COPD = chronic obstructive pulmonary disease, PI = pulmonary impairment. The absence of spontaneous breathing could not be used as indicative of a positive AT result in nine patients because of the detection of relevant levels of analgesics and/or sedatives (sufentanil, fentanyl, midazolam) upon forensic-pharmacological testing. This was also the case in five patients with clinically relevant COPD, [i.e., adaptation to elevated levels of CO_2_, as demonstrated by blood gas analysis: (1) PaCO_2_ outside the required 35–45 mm Hg range, (2) simultaneous pH range of 7.35–7.45, and (3) altered base excess]

### BD/DNC Assessment (Clinical)

BD/DNC testing was performed by two experienced neurointensivists (i.e., board certified in both neurology and intensive care medicine and each having performed > 100 BD/DNC tests) in accordance with the guidelines published by the German Medical Association (Bundesärztekammer) and the German Society of Clinical Neurophysiology. In most cases, BD/DNC testing was required prior to potential organ donation, but in some cases, it was also performed for the purpose of end-of-life care.

For each patient, a thorough review of the case was initially performed, including the examination of all available cerebral imaging and laboratory data. After ruling out alternative factors that could either partly or wholly explain the patient’s comatose state (e.g., sedation, shock, etc.), clinical assessment of brainstem reflexes, including an AT, was performed. A serum drug level for analgesics and/or sedatives was obtained in the following situations: (1) in patients who had received analgesics and/or sedatives (or other centrally acting drugs) for more than 24 h, (2) if the last dosage had been administered < 48 h before BD/DNC testing, and (3) if therapeutic hypothermia (which slows metabolism and medication breakdown) had been applied.

#### AT

The ventilators to which the BD/DNC candidates varied as follows: Dräger Evita4 (*n* = 21), V500 (*n* = 65), V800 (*n* = 33), Evita XL (*n* = 14), Maquet Servo-I (*n* = 2), and Hamilton G5/S1 (*n* = 5). Prior to the AT, the ventilators were set to either Biphasic Positive Airway Pressure (BiPAP) or Synchronized Intermittent Mandatory Ventilation (SIMV) ventilation with a PEEP setting of ≥ 5 mbar (3.75 mm Hg). A rise in PaCO_2_ leads to a drop in pH, which, in turn, results in vasodilatation and loss of blood pressure. To prevent this blood pressure drop during or following the AT, all patients were started on intravenous (IV) noradrenaline treatment (1 or 10 mg/50 mL NaCl 0.9%), that is, if this had not already been administered in the context of general circulatory stabilization. The modified AT (mAT) was performed according to the following standardized protocol:

1. After 10 min of preoxygenation at FiO_2_ 1.0, arterial blood gas analysis and PaCO_2_ measurement (including temperature correction) ensued. The initial target value of PaCO_2_ was 35–45 mm Hg, and ventilation parameters (i.e., respiratory rate, tidal volume) were adjusted if PaCO_2_ levels were outside these limits.
2. PEEP settings were then increased by two points. After checking for any trapped water in the breathing hose that connects the intubation tube to the ventilator, the flow trigger was set to 10 L/min (to prevent triggering of breaths by air shifts in the intubation tube caused by aortic pulsations) and the automatic apnea back-up ventilation was turned off. The acoustic apnea alarm time was set to 60 s. The ventilation mode was then switched to CPAP, and a timer was started.
3. The patient’s thorax and abdomen were undressed and closely monitored for breathing attempts and thorax excursions. Furthermore, the machine display was set to show (a) spontaneous vs. ventilator-induced breaths for automatic recording of resumption of spontaneous breathing and (b) airflow (L/min) to control for pulse-synchronous air flow caused by cardiac/vascular pulsations and to prevent auto-triggering.
4. Blood gas analysis was repeated 6–8 min after switching to CPAP. The AT was deemed positive if PaCO_2_ was ≥ 60 mm Hg and spontaneous breathing was absent. The ventilator was then immediately switched back to pre-AT settings. If temperature-corrected PaCO_2_ was still below 60 mm Hg, the mAT was continued in CPAP mode and blood gas analysis was repeated until either the aforementioned PaCO_2_ target value was reached or spontaneous breathing attempts were witnessed. If spontaneous breathing attempts occurred before the 8-min period had passed, the AT was aborted and the ventilator was switched back to pre-AT settings.

### Patients on Venoarterial ECMO

The mAT was performed in patients on venoarterial ECMO (vaECMO) using the protocol previously described. ECMO settings were adapted as previously described [21]: FiO_2_ to the membrane lung was set to 100%, the sweep gas flow was reduced to 0.5–1.0 L/min, and the pump flow remained unchanged. This was sufficient to achieve a rise in PaCO_2_ in all patients.

### Statistical Analysis and Data Presentation

Statistical analyses (specificity, sensitivity, and positive and negative predictive values) were performed using the IBM SPSS Statistics 21 software package (IBM Corporation, Armonk, NY). Data were found to be nonnormally distributed and are presented as the median with interquartile range. The performance of the mAT was assessed in relation to the extent of PI. The latter was determined by reference to the Horovitz index [22] (arterial partial pressure of oxygen (PaO_2_)/FiO_2_ post 10-min preoxygenation; PEEP was ≥ 5 mbar [3.75 mm Hg] in all patients), and all patients were categorized on this basis: no PI (Horovitz index > 300 mm Hg), mild PI (Horovitz index 201–300 mm Hg), moderate PI (Horovitz index 101–200 mm Hg), and severe PI (Horovitz index ≤ 100 mm Hg) (in analogy to the modified Berlin definition for ARDS).

## Results

Patient characteristics and the feasibility of the mAT are summarized in Table 1.

### Apneic Oxygenation

**Fig. 1:**
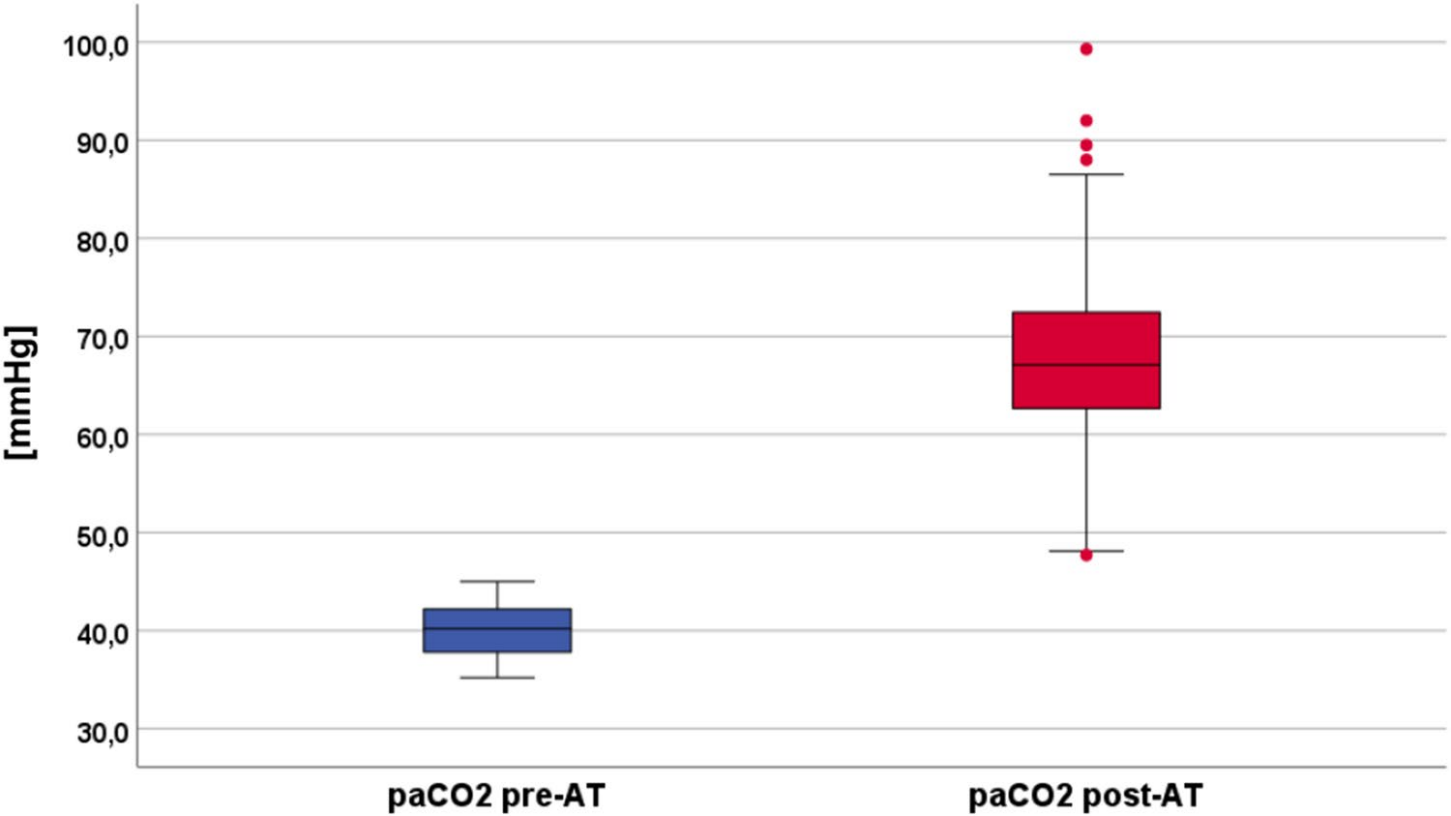
Arterial partial pressure of carbon dioxide (PaCO_2_) values (mm Hg) pre-AT and post-AT. AT apnea test, PaCO_2_

**Fig. 2:**
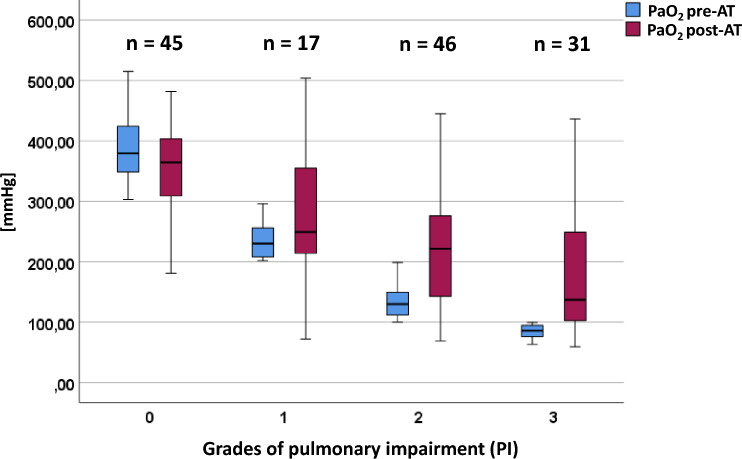
Arterial partial pressure of oxygen (PaO_2_) values pre-AT (after 10-min preoxygenation at 100% fraction of inspired oxygen) versus post-AT. The results are categorized into four groups according to the Horovitz index: 0 = no PI, 1 = mild PI, 2 = moderate PI, and 3 = severe PI (in accordance with the modified Berlin definition for acute respiratory distress syndrome). AT apnea test, PI pulmonary impairment

The PaCO_2_ and PaO_2_ values that were measured pre-AT and post-AT (overall results and PaO_2_ categorized according to the Horovitz index) are shown in Figs. 1 and 2, respectively. The median body temperature at the time of the mAT was 36.3 °C (interquartile range 35.8–36.75 °C).

### Patients on vaECMO

Sixteen patients were under vaECMO treatment at the time of the AT. No significant hypoxemia or desaturation was noted during the mAT in these patients. A rise in PaCO_2_ to at least 60 mm Hg was achieved in all patients solely by reducing sweep gas flow without additionally altering pump flow.

### Complications

#### Deoxygenation

**Fig. 3:**
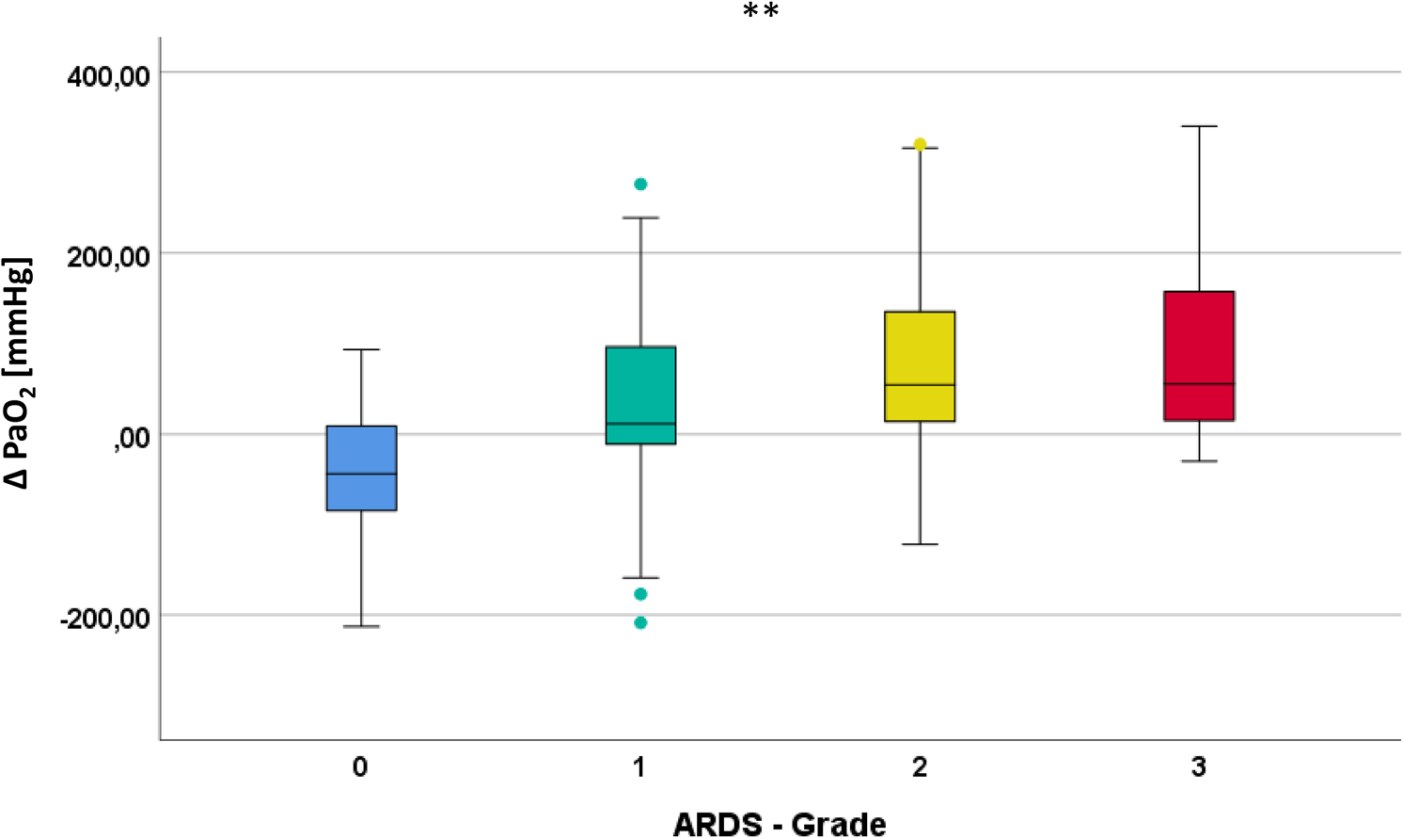
Display of the Δ between PaO_2_ pre-mAT and post-mAT categorized according to the Horovitz index pre-mAT (after 10-min preoxygenation). Grades of PI: 0 = no PI, 1 = mild PI, 2 = moderate PI, and 3 = severe PI. *p < 0.05; **p < 0.01. mAT modified apnea test, arterial partial pressure of oxygen (PaO_2_), PI pulmonary impairment

It was not necessary to either interrupt or abort the mAT (e.g., because of insufficient oxygenation) in any of the 139 patients. The pre-mAT and post-mAT PaO_2_ values are displayed in Fig. 2, subdivided according to the different PI grades (0 [none] to 3 [severe]). Figure 3 illustrates the Δ between pre-mAT and post-mAT PaO_2_ values. No significant difference in Δ values was found in patients without PI. In contrast, the Δ with higher post-mAT PaO_2_ levels was significant in patients with mild PI (*p* < 0.05) and highly significant in patients with moderate and severe PI (*p* < 0.01). When compared with pre-mAT PaO_2_ levels, post-mAT PaO_2_ levels were reduced in 51 patients but were equal or higher in 88 patients. There was no case in which PaO_2_ was ≤ 60 mm Hg in the post-mAT phase (i.e., no mAT-induced hypoxemia was found).

### Cardiocirculatory Issues

In the majority of patients (*n* = 123), blood pressure was found to be lower post-AT than it was prior to the AT. This blood pressure loss occurred in the last minutes or after mAT in all these cases. A total of 104 patients received IV noradrenaline before the mAT was initiated. A noradrenaline perfusor was prepared and connected prior to mAT initiation for the remaining 35 patients. Mean arterial pressure (MAP) transiently dropped below 60 mm Hg in 12 patients, thus requiring the dose of IV norepinephrine to be transiently increased. No other severe complications during the AT were observed.

## Discussion

The AT is a central component of international guidelines for BD/DNC and forms the most critical part of clinical testing because of its potential to harm the patient. Ideally, the AT should be a safe and readily applicable means of testing central respiratory drive status in most patients. Nonetheless, the traditional AT involves disconnection of the patient’s endotracheal tube from the ventilator (with or without additional use of a T-piece or resuscitation bag with CPAP valve) [1, 5], which can result in de-recruitment, desaturation, and early termination. Our study demonstrates not only an alternative approach but also a technical refinement that precludes the occurrence of these detrimental side effects and is easily implemented into intensive care practice. Respiratory failure is particularly frequent in patients with acute severe neurological diseases [10]. Previous studies have reported that execution of the AT was impossible in 4.3–7% of patients because of cardiopulmonary instability and was further aborted in 1.6–3% [23, 24]. In our cohort, only 1 (0.007%) of the 140 patients tested was considered too unstable to undergo the mAT, whereas in the remaining cases, there was no need for the test to be aborted. Indeed, the loss of PEEP that results from disconnection might lead to cardiopulmonary instability, particularly in patients with concomitant severe pulmonary diseases such as ARDS. However, because the method presented here allows the AT to be performed without disconnection from the ventilator, it avoids this potentially critical loss of PEEP and is therefore safer and highly applicable to everyday clinical practice.

The main aim of the AT is to reliably determine whether the patient’s respiratory drive is still intact. In our study, all 12 candidates in whom the AT demonstrated preserved spontaneous breathing showed deep breaths that were similar to the breathing patterns previously described in patients with cerebral lesions [16, 25–27]. These observations were additionally corroborated by the automatic detection of patient-triggered breaths on all the ventilators used in this study.

It is important to consider patients in whom it is impossible to rate the AT as positive. This refers to patients with an absence of spontaneous respiration at PaCO_2_ ≥ 60 mm Hg, either because of a loss of the central respiratory drive or other factors. In this context, many guidelines only refer to PI (e.g., caused by chronic obstructive pulmonary disease or chest contusion) or centrally acting drugs or medication. However, patients with lesions in the upper cervical spinal cord should also be taken into consideration because it is not possible in this cohort to distinguish between central failure of spontaneous breathing caused by brainstem areflexia and partial to complete peripheral failure of spontaneous breathing caused by lesions of the anterior horn neurons (C2–C4) or nerve tracts of the phrenic nerve.

To solve the main problem of ensuring the patient is sufficiently oxygenated (and hereby protecting the patient from AT-induced dangers and facilitating organ protection), the PEEP settings were increased by two points in every case. This allowed adequate alveolar O_2_ circulation and ensured sufficient oxygenation in all patients, despite a median apneic oxygenation duration of 15 min in the absence of ventilation. At the same time, the mere two-point increase in PEEP without ventilation did not result in CO_2_ washout, which permitted the required PaCO_2_ increase to ≥ 60 mm Hg.

Notably, not only was performing the mAT with apneic oxygenation feasible in patients with various extents of PI but it also proved to be favorable: after 10 min of preoxygenation, the patients were classified according to the level of PI (grades 0–3), in accordance with the modified Berlin definition for ARDS [28]. In patients with no PI (grade 0), the Δ value did not significantly differ between pre-mAT and post-mAT PaO_2_. In contrast, patients with mild PI (grade 1) showed a significant increase in post-mAT PaO_2_ (*p* < 0.05), whereas this difference was more pronounced and highly significant (*p* < 0.01) in patients with moderate (grade 2) and severe (grade 3) PI. The most probable explanation for this is that the “apneic oxygenation” phase during alveolar shear stress (cyclic alveolar recruitment and de-recruitment) is omitted during the mAT, which subsequently benefits oxygenation in patients with PI. Moreover, this effect on oxygenation appears to increase incrementally with rising degrees of PI. This result may further promote the use of mAT in preference to the traditional disconnection method, particularly in patients with moderate or severe PI.

The increase in PaCO_2_ is known to cause a possible drop in blood pressure, a phenomenon that was also observed in our cohort. Several underlying mechanisms have been discussed in this respect: (1) a drop in pH during the AT with subsequent vasodilation [29, 30], (2) cardiovascular depression due to direct inhibition of cardiac and smooth muscle cell contractility [31], and (3) an acidosis-induced reduction in the vasoconstrictive effect of catecholamines [32]. Because the drop in blood pressure only ever occurred toward the end of or after the mAT, we attribute the blood pressure drop to hypercarbic vasodilation (and potentially to the reduced effect of catecholamines) rather than to the increased PEEP during the mAT. Norepinephrine was administered in most BD/DNC candidates in our study prior to initiation of the mAT, that is, if it had not already been given to the patient as means of general circulatory stabilization. Specific minimum blood pressure standards for AT initiation (e.g., systolic blood pressure ≥ 100 mm Hg and MAP ≥ 75 mm Hg) are sensible, particularly when using the disconnection method, and can be achieved in most patients before AT initiation [1]. However, in the few patients in our study whose MAP transiently dropped below these thresholds—even to < 60 mm Hg toward the end of or after the mAT—a transient increase in the dose of IV norepinephrine was sufficient to correct this, as previously described [16]. Furthermore, none of the patients experienced critical destabilization of blood pressure, despite the occurrence of high post-mAT PaCO_2_ values in some cases. Cardiocirculatory complications (e.g., arrhythmia, circulatory arrest) have been described previously [11] but did not occur in our cohort. This also held true for the 16 patients in this study who were treated with vaECMO.

Disconnection from the ventilator has been advocated in the past to clearly distinguish auto-triggering, which may falsely be interpreted as a preserved ventilatory drive, from real patient-generated breaths. Because the direct visualization of air flow on the monitor is possible in most existing ventilator models, pulse-synchronous air flow that is caused by aortic or cardiac pulsations can be quantified and the flow trigger can be set accordingly. This enables clear differentiation between machine-generated and patient-generated breaths. Because the mAT could be performed without significant complications in 139 of 140 patients, we suggest omitting disconnection from the ventilator when performing the AT.

Nowadays, there are very few ventilators that do not allow complete a shutdown of apnea ventilation (e.g., two mandatory breaths per minute). In these rare cases, and as an exception, hypoventilation in combination with brief ventilator disconnection (1–2 min) after reaching a PaCO_2_ value of 60 mm Hg could serve as a feasible option, as previously suggested [16]. Machine-generated breaths occur at fixed intervals and can hence be clearly distinguished from patient-generated spontaneous breaths. Nevertheless, particular care needs to be taken to avoid confusion. The disconnection period should be kept as short as possible, as previously described [16].

Because our study was conducted without a control group in a single-center setting and with ventilators that allowed complete shutdown of apnea ventilation, its generalizability is limited. The findings would benefit from additional data generated in a multicenter setting, which is currently being planned on a national level by the Initiative of German NeuroIntensive Trial Engagement (IGNITE!) Network of the German Society of Neurointensive Care. In this project, the control group will consist of patients who undergo a standard AT with disconnection from the ventilator. We discourage the application of the mAT in patients who are on ventilators that allow neither the direct visualization of air flow nor the adaptation of the flow trigger because auto-triggering cannot be adequately controlled for in this case. In ventilators that do not enable a complete shutdown of apnea back-up ventilation, particular care needs to be taken to not confuse machine-generated and patient-generated breaths. Because the vast majority of ventilators in our study did allow complete shutdown of apnea back-up ventilation, this may represent a potential limitation.

## Conclusions

We have described a modified version of the AT that does not require disconnection from the ventilator and is performed in combination with an increase in PEEP. This mAT represents a safe and easy means of determining central respiratory drive status, particularly in patients with PI.

## References

[1] Kirschen MP, Lewis A, The GDM. American academy of neurology, american academy of pediatrics, child neurology society, and society of critical care medicine pediatric and adult brain death/death by neurologic criteria determination consensus guidelines: what the critical care team needs to know. Crit Care Med. 2023. 10.1097/CCM.0000000000006099. 37921516 10.1097/CCM.0000000000006099

[2] Montgomery FU, Scriba PC, Tonn J-C and Angstwurm H. Richtlinie gemäß § 16 Abs. 1 S. 1 Nr. 1 TPG für die Regeln zur Feststellung des Todes nach § 3 Abs. 1 S. 1 Nr. 2 TPG und die Verfahrensregeln zur Feststellung des endgültigen, nicht behebbaren Ausfalls der Gesamtfunktion des Großhirns, des Kleinhirns und des Hirnstamms nach § 3 Abs. 2 Nr. 2 TPG, Vierte Fortschreibung. (Vierte Fortschreibung).

[3] Silvester W, Bevan R, Brieva J, Cook D, D’Costa R, Dobb G, Gelbart B, Jones S, Judson J, Modra L, Moodie S, Opdam H, Poynter C, Streat S. The Statement on Death and Organ Donation. Edition 4.1. Australian and New Zealand Intensive Care Society (ANZICS); 2021.

[4] Simpson P, Bates D, Bonner S, Costeloe K, Doyal L, Falvey S, Gaffin J, Howard R, Kane N, Kennedy CR, Kennedy I, Kerr S, Manara A, Pickard J, Rolles K, Short A. A code of practice for the diagnosis and confirmation of death. Academy of Medical Royal Colleges; 2008.

[5] Greer DM, Shemie SD, Lewis A, Torrance S, Varelas P, Goldenberg FD, et al. Determination of brain death/death by neurologic criteria: the world brain death project. JAMA. 2020;324(11):1078–97. 10.1001/jama.2020.11586. 32761206 10.1001/jama.2020.11586

[6] Wijdicks EFM, Varelas PN, Gronseth GS, Greer DM. American Academy of Neurology: Evidence-based guideline update: determining brain death in adults: report of the Quality Standards Subcommittee of the American Academy of Neurology. Neurology. 2010;74(23):1911–8. 20530327 10.1212/WNL.0b013e3181e242a8

[7] Schwarz G, Errath M, Arguelles Delgado P, Schöpfer A, Cavic T. Ventilator autotriggering : an underestimated phenomenon in the determination of brain death. Anaesthesist. 2019;68(3):171–6. 30810759 10.1007/s00101-019-0555-5

[8] Yee AH, Mandrekar J, Rabinstein AA, Wijdicks EF. Predictors of apnea test failure during brain death determination. Neurocrit Care. 2010;12(3):352–5. 20217276 10.1007/s12028-010-9343-4

[9] Hocker S, Whalen F, Wijdicks EFM. Apnea testing for brain death in severe acute respiratory distress syndrome: a possible solution. Neurocrit Care. 2014;20(2):298–300. 24233817 10.1007/s12028-013-9932-0

[10] Mascia L, Sakr Y, Pasero D, Payen D, Reinhart K, Vincent JL, et al. Extracranial complications in patients with acute brain injury: a post-hoc analysis of the SOAP study. Intensive Care Med. 2008;34(4):720–7. 10.1007/s00134-007-0974-7. 18175107 10.1007/s00134-007-0974-7

[11] Goudreau JL, Wijdicks EF, Emery SF. Complications during apnea testing in the determination of brain death: predisposing factors. Neurology. 2000;55(7):1045–8. 11061269 10.1212/wnl.55.7.1045

[12] Migdady I, Shoskes A, Amin M, Cho SM, Rae-Grant A, George P. Determination of brain death in patients undergoing short-term mechanical circulatory support devices. Heart Lung Circ. 2022;31(2):239–45. 34210616 10.1016/j.hlc.2021.05.100

[13] Giani M, Scaravilli V, Colombo SM, Confalonieri A, Leo R, Maggioni E, et al. Apnea test during brain death assessment in mechanically ventilated and ECMO patients. Intensive Care Med. 2016;42(1):72–81. 10.1007/s00134-015-4105-6. 26556611 10.1007/s00134-015-4105-6

[14] Lévesque S, Lessard MR, Nicole PC, Langevin S, LeBlanc F, Lauzier F, et al. Efficacy of a T-piece system and a continuous positive airway pressure system for apnea testing in the diagnosis of brain death. Crit Care Med. 2006;34(8):2213–6. 16540953 10.1097/01.CCM.0000215114.46127.DA

[15] Kramer AH, Couillard P, Bader R, Dhillon P, Kutsogiannis DJ, Doig CJ. Prevention of hypoxemia during apnea testing: a comparison of oxygen insufflation and continuous positive airway pressure. Neurocrit Care. 2017;27(1):60–7. 28176180 10.1007/s12028-017-0380-0

[16] Lang CJG, Heckmann JG. Apnea testing for the diagnosis of brain death. Acta Neurol Scand. 2005;112(6):358–69. 16281917 10.1111/j.1600-0404.2005.00527.x

[17] Gutmann DH, Marino PL. An alternative apnea test for the evaluation of brain death. Ann Neurol. 1991;30(6):852–3. 10.1002/ana.410300620. 1789700 10.1002/ana.410300620

[18] Benzel EC, Mashburn JP, Conrad S, Modling D. Apnea testing for the determination of brain death: a modified protocol. Technical note J Neurosurg. 1992;76(6):1029–31. 1588410 10.3171/jns.1992.76.6.1029

[19] Solek-Pastuszka J, Sawicki M, Iwańczuk W, Bohatyrewicz R. Ventilator-delivered continuous positive airway pressure for apnea test in the diagnosis of brain death in patient with extremely poor baseline lung function—case report. Transplant Proc. 2016;48(7):2471–2. 27742325 10.1016/j.transproceed.2016.02.089

[20] Ahlawat A, Carandang R, Heard SO, Muehlschlegel S. The modified apnea test during brain death determination: an alternative in patients with hypoxia. J Intensive Care Med. 2016;31(1):66–9. 26574562 10.1177/0885066615599086 PMC4684955

[21] Winter S, Groesdonk HV, Beiderlinden M. Apnea test for assessment of brain death under extracorporeal life support. Med Klin Intensivmed Notfallmedizin. 2019;114(1):15–20. 10.1007/s00063-017-0287-8 28444410

[22] Horovitz JH, Carrico CJ, Shires GT. Pulmonary Response to Major Injury. Arch Surg. 1974;108(3):349–55. 10.1001/archsurg.1974.01350270079014. 4813333 10.1001/archsurg.1974.01350270079014

[23] Wijdicks EFM, Rabinstein AA, Manno EM, Atkinson JD. Pronouncing brain death: contemporary practice and safety of the apnea test. Neurology. 2008;71(16):1240–4. 18852438 10.1212/01.wnl.0000327612.69106.4c

[24] Datar S, Fugate J, Rabinstein A, Couillard P, Wijdicks EFM. Completing the apnea test: decline in complications. Neurocrit Care. 2014;21(3):392–6. 24522760 10.1007/s12028-014-9958-y

[25] Wijdicks EFM. Biot’s breathing. J Neurol Neurosurg Psychiatry. 2007;78(5):512–3. 17435185 10.1136/jnnp.2006.104919 PMC2117832

[26] Summ O, Hassanpour N, Mathys C, Groß M. Disordered breathing in severe cerebral illness: towards a conceptual framework. Respir Physiol Neurobiol. 2022;300: 103869. 35181538 10.1016/j.resp.2022.103869

[27] Whited L, Hashmi MF, Graham DD. Abnormal Respirations. In: StatPearls. Treasure Island (FL): StatPearls Publishing; 2023 [cited 2023 Nov 28]. Available from: http://www.ncbi.nlm.nih.gov/books/NBK470309/29262235

[28] ARDS Definition Task Force, Ranieri VM, Rubenfeld GD, Thompson BT, Ferguson ND, Caldwell E, et al. Acute respiratory distress syndrome: the Berlin Definition. JAMA. 2012;307(23):2526–33. 22797452 10.1001/jama.2012.5669

[29] Lang CJG. Blood pressure and heart rate changes during apnoea testing with or without CO2 insufflation. Intensive Care Med. 1997;23(8):903–7. 10.1007/s001340050430. 9310811 10.1007/s001340050430

[30] Scott JB, Gentile MA, Bennett SN, Couture M, MacIntyre NR. Apnea testing during brain death assessment: a review of clinical practice and published literature. Respir Care. 2013;58(3):532–8. 22709413 10.4187/respcare.01962

[31] Crystal GJ. Carbon dioxide and the heart: physiology and clinical implications. Anesth Analg. 2015;121(3):610. 26287294 10.1213/ANE.0000000000000820

[32] Schotola H, Toischer K, Popov AF, Renner A, Schmitto JD, Gummert J, et al. Mild metabolic acidosis impairs the β-adrenergic response in isolated human failing myocardium. Crit Care. 2012;16(4):R153. 22889236 10.1186/cc11468 PMC3580742

